# Towards clinical application of implantable brain–computer interfaces for people with late-stage ALS: medical and ethical considerations

**DOI:** 10.1007/s00415-022-11464-6

**Published:** 2022-11-30

**Authors:** Mariska J. Vansteensel, Eran Klein, Ghislaine van Thiel, Michael Gaytant, Zachary Simmons, Jonathan R. Wolpaw, Theresa M. Vaughan

**Affiliations:** 1 Department of Neurology and Neurosurgery, UMC Utrecht Brain Center, University Medical Center Utrecht, P.O. Box 85060, 3508 AB Utrecht, The Netherlands; 2 Department of Neurology, Oregon Health and Science University, Portland, OR USA; 3 Department of Philosophy, University of Washington, Seattle, WA USA; 4 Julius Center for Health Sciences and Primary Care, Department Medical Humanities, University Medical Center Utrecht, Utrecht, The Netherlands; 5 Department of Pulmonary Diseases/Home Mechanical Ventilation, University Medical Center Utrecht, Utrecht, The Netherlands; 6 Department of Neurology, Pennsylvania State University, Hershey, PA USA; 7 National Center for Adaptive Neurotechnologies, Albany Stratton VA Medical Center, Department of Biomedical Sciences, State University of New York, Albany, NY USA; 8 National Center for Adaptive Neurotechnologies, Albany Stratton VA Medical Center, Albany, NY USA

**Keywords:** Brain–computer interface, Implant, Amyotrophic lateral sclerosis, Ethics, Clinical application, Tracheostomy invasive ventilation

## Abstract

Individuals with amyotrophic lateral sclerosis (ALS) frequently develop speech and communication problems in the course of their disease. Currently available augmentative and alternative communication technologies do not present a solution for many people with advanced ALS, because these devices depend on residual and reliable motor activity. Brain–computer interfaces (BCIs) use neural signals for computer control and may allow people with late-stage ALS to communicate even when conventional technology falls short. Recent years have witnessed fast progression in the development and validation of implanted BCIs, which place neural signal recording electrodes in or on the cortex. Eventual widespread clinical application of implanted BCIs as an assistive communication technology for people with ALS will have significant consequences for their daily life, as well as for the clinical management of the disease, among others because of the potential interaction between the BCI and other procedures people with ALS undergo, such as tracheostomy. This article aims to facilitate responsible real-world implementation of implanted BCIs. We review the state of the art of research on implanted BCIs for communication, as well as the medical and ethical implications of the clinical application of this technology. We conclude that the contribution of all BCI stakeholders, including clinicians of the various ALS-related disciplines, will be needed to develop procedures for, and shape the process of, the responsible clinical application of implanted BCIs.

## Introduction

Following a diagnosis with amyotrophic lateral sclerosis (ALS), progressive motor impairment compels affected individuals to make minor and major decisions related to their care, treatment goals, assistive and life-sustaining technology and, usually, end-of-life wishes [1]. Given the frequently fast progression of the disease and the current lack of curative treatment options, these decisions typically relate to improving or maintaining quality of life, and therefore essentially fall in the area of (neuro)palliative care, from the time of diagnosis onwards [2].

**Fig. 1:**
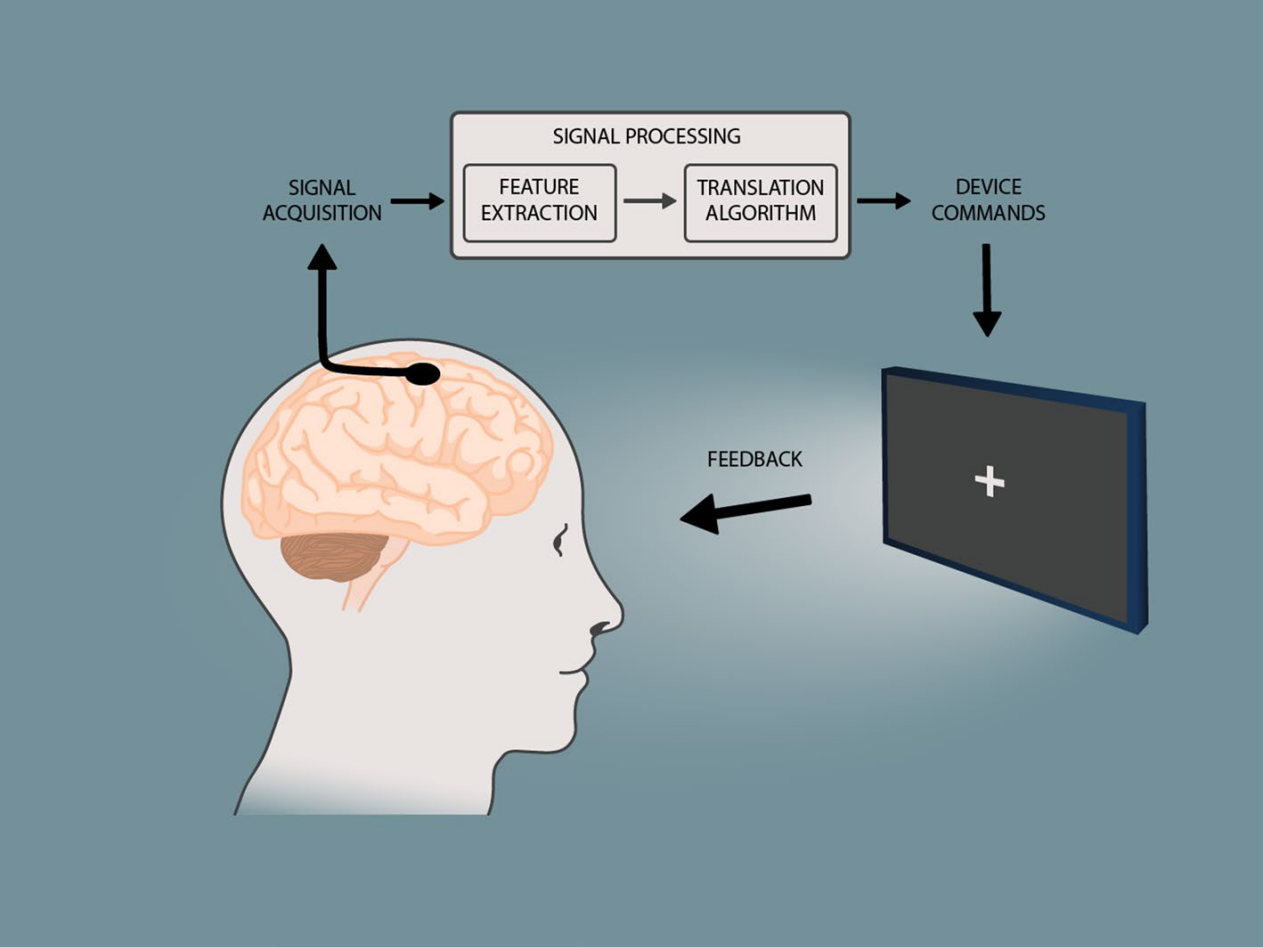
Schematic representation of the concept of a brain–computer interface. Neural signals are read from the brain using electrodes or other sensors. In this article, we focus on electrodes that are implanted on, or in, the brain. Specific features that reflect the intention of the user are extracted from the acquired signals, and translated into a command to control an application, such as communication software on a computer

A central theme in the management of ALS is helping individuals address the loss of the ability to communicate verbally or in writing, related to the progressive motor impairment and the use of tracheostomy invasive ventilation (TIV). Conventional assistive communication technologies rely on some level of residual and intentional muscle movement for control and thus do not represent an adequate solution for all people with ALS (PALS) in all disease stages and all circumstances [3–6]. Brain-computer interface (BCI) technology is a novel, muscle-independent method to control computers and communication software, using brain signals. BCIs rely on a feedback loop where neuroelectric signals are recorded from the brain with electrodes placed on the scalp (non-implanted BCI) or surgically implanted *on* or *in* the brain (implanted BCI), followed by the extraction of specific features from the acquired signals, and the translation of these features into a control signal for a computer (Fig. 1). In the past several years, the BCI field has made significant progress, and the first demonstrations of successful home use of non-implanted and implanted BCIs specifically for communication by people with severe motor impairment, including PALS, have appeared [7–11]. As such, BCIs are considered a promising tool for allowing PALS to continue communicating in cases, circumstances or ALS disease stages where conventional assistive technology falls short. In addition, there is increasing attention being paid to a range of factors affecting whether a BCI is considered usable and will be embraced by the severely motor-impaired target population. This determination hinges on effectiveness (e.g., accuracy), efficiency (e.g., information transfer rate, workload), and user and caregiver satisfaction (e.g., for use in daily life) [12, 13].

Although BCIs are still largely confined to the research domain, the current pace of technological and neuroscientific development leaves no doubt that BCIs will enter the clinical realm at some point in the future, and become available for clinicians to provide to PALS who face losing, or who have lost, the ability to communicate. Upon clinical application, BCIs may profoundly affect the daily lives and clinical care of PALS. This is especially relevant in relation to *implanted* BCIs, as the surgery that is needed to implant the neural signal recording electrodes may interact with other medical procedures PALS undergo (such as TIV surgery), with respect to timing and risks of the surgery, as well as considerations about quality of life and decisions about end-of-life.

This manuscript aims to contribute to a responsible clinical application of implanted BCIs for communication in PALS, by drawing attention to the need for increasing the engagement of clinicians involved in the management of ALS in the continued research, development, and application of this technology. To that end, we discuss current knowledge about the quality of life of PALS, and consider how this can be affected by two important, and sometimes interrelated factors, namely respiratory support and the ability to communicate. Then we review the current state of the art of implanted BCIs for communication, as well as medical and ethical issues that require attention before PALS can start to benefit widely from implanted BCIs. We conclude with a call for a multidisciplinary approach in all research and development steps aimed at the clinical implementation of implanted BCIs.

## Quality of life in ALS

Quality of life (QoL) as perceived by people with ALS (PALS) has been widely investigated. Importantly, findings on the relation between QoL and the level of functional impairment or disease stage are affected by differences in the assessment tools used. Tools that focus on *health-related* QoL, which represent health status and physical ability, typically show a worsening of QoL as the disease progresses [14–16]. On the other hand, studies that use QoL assessment tools that focus more on the perspective of the individual and their overall, subjective wellbeing have revealed that a positive QoL can be maintained despite disease progression [14, 17, 18]. Indeed, QoL is often reported as high by PALS with severe motor impairment [19, 20] and such *overall* QoL may even be comparable to QoL in healthy controls [17]. These observations are thought to reflect an adaptation process that changes the QoL perspective of individuals with severe health problems, a phenomenon called ‘response shift’ [21].

Interestingly, the general public, as well as caregivers of PALS and physicians with little experience with ALS and palliative care, may not be sufficiently aware of the relatively high rating of their own QoL by PALS: several studies have shown that these groups may underestimate QoL perceived by PALS [19, 22–24]. Perhaps relatedly, not all neurology residents receive formal training in palliative care issues [25–27], and therefore PALS may be treated by clinicians without sufficient experience with these topics. Underestimation of the QoL of PALS by the general public, on the other hand, may reflect the fact that they usually have little exposure to people with ALS and judge QoL from their own perspective. In contrast, underestimation of the QoL of PALS by their caregivers may reflect the caregivers’ own sense of wellbeing [23], which is frequently low due to their high levels of physical and emotional burden (see for review: [28, 29]).

## Respiratory support for people with ALS

Average survival of PALS from onset of symptoms is about 2–3 years, although some survive much longer [30–32]. Recent advances in the field of ventilator technology, however, now allow PALS to receive non-invasive ventilation (NIV) or TIV in their home (home mechanical ventilation; HMV). This has proved to be an effective treatment strategy for patients with chronic respiratory failure, including PALS, with significant extension of survival [33–39]. Not surprisingly, the prevalence of HMV continues to rise [40] due to advances in diagnostic and supportive technology, improved health-care delivery, and better understanding of the beneficial effects on QoL and potential cost savings to health-care systems [41].

PALS use of NIV and TIV shows notable regional differences [42]. TIV use is relatively low in the US (6% [43]) and in several European and Asian countries (UK 0% [44]; The Netherlands 1.3% [45]; Germany 3.3–9.5% [39, 44]; Norway/Sweden 3.8–6.7% [46]; Korea 8.9% [47]; Italy 10.6% [48]). TIV use by PALS is much more common in Japan (around 30% [49, 50]) and Denmark (~ 22% [36]). The reasons for these differences are not entirely clear; they may reflect psychosocial factors and different balances between the advantages and disadvantages of TIV [39] or different attitudes towards terminating mechanical ventilation [42]. In addition, differences across countries in the organization and reimbursement of health care, as well as cultural and religious/spiritual issues, are likely to play a role. It is interesting to note that the use of TIV among PALS seems on the rise in both Japan and Germany [39, 50].

Notably, many tracheostomies in PALS are unplanned and performed in an emergency health crisis [51]. For those who have the opportunity to discuss TIV in advance with their clinicians, the main reasons to start TIV are problems with NIV due to bulbar symptoms or the wish to live as long as possible. In those cases, the decision-making process is typically affected by factors such as survival, QoL, and the risk of progression to the locked-in state [﻿^52^].

### Respiratory support and QoL

Besides having an effect on survival, NIV may improve the QoL of PALS [33, 53]. The effects of TIV on QoL have been less well studied, most likely due to the relatively low number of PALS receiving TIV worldwide. Available results indicate, however, that TIV can improve QoL [15], that QoL of PALS with TIV is acceptable [54, 55], and that most PALS with TIV have positive views about it [55, 56]. More research is needed to assess the effects of TIV on QoL, taking into account for example the effects of living environment (at home versus hospitalized) and TIV initiation (emergency versus elective).

## Communication in ALS

Due to bulbar symptoms or the use of TIV with a cuffed tube, patients can lose their ability to communicate via speech, which, in combination with ALS-induced tetraplegia, in essence represents a locked-in syndrome (LIS; defined by sustained eye opening, severe hypophonia or aphonia, quadriparesis, evident cognitive abilities, and the use of eye movement or blinking for communication (American Congress of Rehabilitation Medicine, [57])). Depending on the level of functional impairment and personal preferences, people with ALS can use different no-, low- or high-tech augmentative and alternative communication (AAC) solutions [58]. Importantly, these conventional solutions all rely on some level of residual and functional movement. No-tech solutions, for example, include making eye blinks in response to closed questions, or to letters recited one-by-one by a communication partner. Low-tech solutions comprise, among others, pen and paper, letter cards, and word books. High-tech solutions involve an electronic device or computer that can pronounce text entered by the user. Text entry can be accomplished in several ways (access methods), such as with a mouse or joystick controlled by reliable movements of a foot, hand, tongue or the eyes. When residual movements are only small and one dimensional, they activate a switch for control of communication programs in *switch scanning mode*. During switch scanning, different fields of a matrix of letters, words or icons are highlighted automatically and sequentially and individual fields are selected by correctly timed switch signals generated by the user.

A high-tech solution that is particularly valuable for people with advanced ALS is an eye-gaze device, which detects and tracks eye movements towards letters, words or icons on a computer screen and allows for relatively fast communication. Since eye movements are typically spared until late very stages of ALS, eye gaze is often the only usable, muscle-based, method for computer access for people with advanced ALS, including those who receive TIV [4, 5]. Eye-gaze devices are often successful [3] and the technology is considered useful by many PALS, also in late stages of the disease [59, 60]. Importantly, also from the perspective of caregivers, eye-gaze devices are considered helpful, because they often decrease caregiver burden and increase quantity and quality of communication [59].

Despite the positive reports about eye-gaze devices, an important subset of people with late-stage ALS have difficulty using this technology [3–5] and about 10–17% of PALS cannot communicate at all [5, 6]. The problems that impair or prevent the use of eye-gaze devices include difficulty maintaining stable head position [4], pupil dilation due to Baclofen use [61], and progressive oculomotor impairment and eye-gaze fatigue [4, 5]. Indeed, and in contrast to the common idea that eye motility is spared in ALS, a substantial number of PALS experience some level of oculomotor impairment, even during early stages of the disease [62–68]. One study reported that ~ 18% of PALS receiving TIV for more than 5 years lost all voluntary motor function, including eye movement (complete locked-in syndrome), and ~ 33% developed a ‘minimal communication state’ [69].

### Communication and QoL

Although the general level of physical impairment does not correlate with overall QoL, losing the ability to speak has significant negative effects on QoL of PALS [70]. In addition, the use of AAC technology, which allows PALS to communicate with their caregivers, participate in family life and retain autonomy, was shown to improve both global and health-related QoL [71–74]. These results underline the crucial role that communication plays in human life and stress the importance of supporting PALS in maintaining communication capabilities at the highest level possible at all stages of the disease. In fact, the “*basic right [of all people, regardless of the extent or severity of their disabilities] to affect, through communication, the conditions of their own existenc*e” has been formalized in a “Communication Bill of Rights” (Brady et al. [75]; National Joint Committee for the Communicative Needs of Persons with Severe Disabilities,” [76]). Given the fact that TIV HMV is available in many countries worldwide and is even on the rise in some countries [39, 50], it can be expected that a significant number of PALS will progress into (very) late stages of the disease. At the same time, for PALS to maintain their communication-related QoL into these late disease stages, where significant oculomotor problems may render eye-gaze devices useless, it will be crucial that alternative, muscle-independent, communication strategies be developed.

### Implantable brain–computer interfaces for communication in late-stage ALS

The most promising approaches for usable brain–computer interface (BCI) solutions employ *neuro-electrical* signals from the brain, which can be recorded with either non-implanted (electroencephalography; EEG) or implanted electrodes. Both recording methodologies show great promise for resolving the communication impairments of PALS, with demonstrations of successful use in settings of daily living [7–11]. Since non-implanted and implanted BCIs each have their own advantages and disadvantages, the importance of which will be weighed differently by different people, each of these two signal recording approaches are likely to eventually serve their own target (sub)populations, similar to how glasses, contact lenses, and laser eye surgery serve different groups of people with vision problems. Thus, the further development, validation, and clinical implementation of both these BCI methods deserve careful attention. Because of the significant medical implications of BCI implantation surgery, we focus here on implanted BCIs. The two most commonly used types of implanted electrodes for BCI purposes are intracortical microelectrode arrays and electrocorticographic (ECoG) electrodes placed on the surface of the brain.

#### Intracortical electrodes

Typically, intracortical electrodes are organized in microelectrode arrays containing about 100 small needles (~ 1 mm in length [77]) that penetrate the cortical surface. High sampling rate recordings of microelectrodes detect the activity of single neurons. Since single neurons in the motor areas are known to be *tuned* to specific movement intentions [78], such as movement direction, and since this tuning may be different for different neurons, recordings with these arrays provide rich information about intended movements, also in people with motor impairment [79]. The concept of directional tuning has been used in studies on the control of virtual keyboards for spelling of words and texts by people with severe motor impairment, including two individuals with ALS [80–83], where typing speeds of several words/min were achieved. More recently, neural activity recorded with intracortical electrodes from the sensorimotor hand area has been used to decode, with high accuracy (> 90%) and speed (up to 90 characters/min), which letter a participant with spinal cord injury attempted to write [84]. Signals with intracortical electrodes have also allowed an individual without any remaining muscle movement (complete locked-in syndrome) due to ALS to spell words and sentences [85].

One BCI-based communication strategy that has been tested with intracortical electrodes is *speech decoding*: determining which sound or word someone pronounces—or tries to pronounce— based on neural signals only. A natural electrode target area for speech decoding is the ventral part of the motor cortex, which is known to be associated with mouth and speech articulation movements. Initial reports on the decoding of vowels and phonemes were obtained with electrodes implanted in that area in an individual who was severely motor impaired by brainstem stroke [86, 87]. More recently, it was shown that neurons in the dorsal part of the sensorimotor cortex, in the area typically associated with hand movements, are also activated during overt speech in people with spinal cord injury [88]. First investigations on the use of this activity to distinguish which word or sound was uttered have provided promising results [88, 89].

Although the scientific accomplishments in the area of communication speed and accuracy obtained with intracortical electrodes are impressive, other factors that determine BCI usability for the target population [12] need to be taken into account. One important factor to address with respect to intracortical electrodes is reliability. It is commonly acknowledged that the neural signals recorded with intracortical electrodes are relatively unstable, showing variance in the course of hours, days, and weeks [81, 90–93]. This variability impairs decoding accuracy and necessitates frequent calibration. Several strategies have been developed to address this problem, including the use of local field potentials (LFPs) instead of single unit recordings [91] and self-calibration of the system [81, 94], which have delivered interesting results in terms of calibration-free recordings over tens of days. A second factor is longevity of the signals from intracortical electrodes. Reports on the longevity of intracortically recorded signals from humans are scarce, but the available evidence suggests that signal quality degrades over the course of time [95]. Nevertheless, a human participant with tetraplegia due to a brainstem stroke [96, 97] and a participant with spinal cord injury [95] were able to generate usable signals for at least 5 years after implantation. Third, while human studies of intracortical BCIs for communication in people with severe motor impairment (including several PALS) have shown the feasibility of the approach, independent use for communication by the target population in daily-life settings, and the user satisfaction associated with that, have yet to be adequately studied.

#### Electrocorticography electrodes

Electrocorticography (ECoG) recordings use silicone strips or grids containing small metal disk-like contact electrodes that are typically placed on the cortical surface (subdurally). Clinically, ECoG is an important diagnostic tool in the presurgical evaluation of epilepsy patients for localization of the epileptic focus and of essential brain function, often using 1 cm-spaced electrode arrays. Important findings obtained with this population include the first demonstrations of the use of voluntarily generated changes in the frequency-domain signals from the sensorimotor cortex (e.g. [98]) or cognitive control areas [99] for upward and downward control of a cursor on a computer screen, as well as the use of visually evoked P300 potentials for selection of letters within a so-called P300 matrix speller [100, 101]. Much of the current BCI research involving people with epilepsy is focused on increasing the number of BCI control signals that are extracted from the brain, often using high spatial density ECoG grids (interelectrode distance on the order of several mm). These efforts, which predominantly rely on signals from the sensorimotor cortex, aim to distinguish, for example, different types of hand movements [102–107], or the pronunciation of different words or sounds (e.g. [108–112]), based on the regions in the sensorimotor cortex that control hand or speech articulation movements, respectively.

So far, attempts to validate ECoG-BCI approaches for communication by people with severe motor impairment have been scarce. Several groups have demonstrated ECoG-BCI control by individuals with tetraplegia due to stroke, spinal cord injury or other events [113–117]. These individuals were able to control a cursor, a robotic hand or an exoskeleton using (attempted) movements of different parts of the upper limb or the head. Recently, Edward Chang and coworkers [118], using signals from a 128-channel, high spatial density ECoG grid implanted in an individual with spastic quadriparesis and anarthria, showed they were able to decode individual words that the participant attempted to pronounce with an accuracy of 41.7% (50-word vocabulary; chance level 2%). When a language model was applied, sentences were decoded at a rate of 15.2 words per minute and with a word error rate of 25.6%.

The first demonstration of independent home use of an implanted ECoG-based BCI system for communication by an individual with late-stage ALS appeared in 2016 (Utrecht Neural Prosthesis, UNP; [9]). The fully implanted, and therefore invisible, BCI system included subdural ECoG strips that were implanted through small burr holes in the skull and connected by subcutaneous leads to an implanted amplifier/transmitter device placed subcutaneously in the chest area. Using attempted movements of the right hand, the user was able to produce ‘brain-clicks’ for the selection of letters or words in a communication program in switch-scanning mode with high (89%) accuracy. Communication speed was limited (2–3 characters/min), largely due to the waiting involved in the use of the switch-scanning interface. Despite that, user satisfaction was high [9, 119]. Importantly, the BCI control signal has been stable over years after implantation [119], and as of today (2022), she still uses the system in her daily life.

#### Other signal acquisition approaches

Two alternative implanted brain signal recording techniques have recently gained attention within the BCI field: stereo-electroencephalography (S-EEG; depth electrodes) and endovascular electrodes.

S-EEG ﻿is a technique to measure from deep structures in the brain. The recording electrodes are arranged along shafts of ~1 mm in diameter (8-18 electrodes per shaft, ~2 mm in length). The technique is increasingly common in presurgical evaluation of people with epilepsy. The ability to reach deeper brain structures and the lower surgical risk compared to ECoG-grid implantation are some of the attractive features of S-EEG. In the past years, several studies have demonstrated the feasibility of decoding motor-related and other signals with S-EEG electrodes (see for review [120]). As far as we are aware, S-EEG has not been applied in PALS or in people with severe motor impairments of other origins.

An entirely new concept for neural signal acquisition for BCI purposes is endovascular brain signal recording. This, so-called ‘Stentrode’ technology is based on venous sinus stents that are clinically applied in the treatment of intracranial hypertension. Similar stents equipped with embedded electrodes enable minimally invasive placement of recording electrodes near cortical areas through the cerebral vasculature [121]. The approach, in which intravenous electrodes placed in the superior sagittal sinus are connected via subcutaneous leads to an implanted telemetry device, has so far been validated with two PALS [11]. Both participants were able to produce a reliable brain-click and used the system in their homes, in combination with their eye-gaze devices, for computer control.

## Towards clinical application of implantable BCIs

### Translational steps

Despite the recent accomplishments with implanted BCIs and the fact that people with motor impairments are starting to benefit from them, several issues need to be addressed in the coming years before implanted BCIs can be widely used clinically.

First and foremost, the usability of implanted BCIs for daily-life communication needs to be assessed with more end users [122]. This effort will not only inform us on specific user characteristics that benefit or impair adequate BCI control, but will also contribute to further, user-centered, development of communication-BCI solutions for daily-life settings. Crucially, such validation efforts need to include, besides PALS, their caregivers, health-care professionals, and other support personnel, and may benefit from a greater availability of funding for studies that replicate previous investigations and at the same time assess factors that support or inhibit BCI use.

Second, to make possible thorough validation of implanted BCIs in settings of daily living, and eventual wide clinical application of these devices, fully implantable recording systems that enable reliable recording and transmission of signals (ideally from many electrodes and at high rates) are essential. For intracortical electrodes, development of wireless solutions has been underway for many years [123–126]; recently, initial reports described human use of portable and wireless intracortical BCI systems [127, 128]. Importantly, the UNP [9], the Stentrode [11], and the epidural WIMAGINE system [113], are fully implantable amplifier–transmitter devices. Unfortunately, none of these systems are widely available at present, nor are other fully implantable devices suitable for BCI purposes; this reality currently prevents large-scale validation of implanted BCI approaches in PALS. It will be interesting to witness further developments in the area of fully implanted and wireless BCIs in the coming years, especially because several large companies have recently expressed interest in this field.

### Medical and ethical considerations

While addressing the abovementioned topics may take some time, the BCI field is maturing rapidly and implanted BCIs are expected to enter the clinical realm in the not-to-distant future. At that point, it will be important to have dedicated procedures and standards of care in place. Some of the topics that deserve attention in the development of these procedures are primarily medical or ethical in nature. Here, we discuss *interaction with TIV; proportionality and responsible use; informed consent for BCI research and treatment;* and *access and continued use*.

#### Interaction with TIV

Future clinical decision-making processes for implanted BCIs need to acknowledge that considerations of PALS regarding TIV and implanted BCIs are related. Those who desire to continue living beyond the point of respiratory failure will need alternative communication strategies, since they are likely to reach stages of the disease where muscle-based control of communication technology becomes increasingly difficult [5]. In turn, the decision about whether or not to choose TIV is often affected by ideas about the future QoL, the ability to communicate, and the risk of reaching the locked-in state [52, 129]. Clinical availability of implanted BCIs is likely to influence the patients’ outlook on future QoL and thoughtful attention for their needs and wishes, and those of the patients’ support systems is called for. From a medical point of view, the timing and the potential risks of the required surgical interventions may apply to both TIV and implantable BCIs. Therefore, the clinical application of implantable BCIs will require development of procedures that aim to inform *all* PALS, in a timely fashion, about the possibilities and consequences of receiving TIV and implanted BCIs, so that they can engage in a voluntary and well-informed decision-making process concerning these technologies.

#### Proportionality and responsible use

A key aspect of responsible use of implanted BCIs in PALS is proportionality, meaning a favorable balance between the potential benefits on the one hand and the risks and burdens on the other. Further and more widespread introduction of BCIs should be accompanied by careful assessment of aspects beyond those typically addressed for regulatory approval of medical devices (efficacy and safety); these important additional aspects include accuracy, reliability, usefulness, usability, and privacy. There are also profound philosophical issues, for example, related to the concept of self and personhood [130]. We will not discuss the latter here, but instead address several aspects that go beyond the scope of traditional risk–benefit assessments and that are especially relevant for implanted BCIs in PALS.

First, to achieve optimal benefits of BCIs, it may at first seem logical to offer the treatment to PALS close to or after the moment that functional communication is lost. However, studies mainly among patients undergoing deep brain stimulation for Parkinson’s disease have revealed that a significant number of patients experience post-operative psychological and social burdens related to restoration of previously lost functions. The phenomenon is known as the “burden of normality” [131, 132]. In addition, people with neuromuscular disease vary significantly as to when they become interested to—or perhaps even willing to—discuss the difficult and complex issue of whether to use an implanted BCI to maintain communication. Some people prefer to be informed shortly after the initial diagnosis of ALS; others only want to consider assistive technology, including BCIs, when they become viable options for maintaining communication and control [133]. This raises the question of optimal timing to offer the option of BCI to patients with ALS.

Second, the possible side effects of BCI technology on patients’ autonomy are not clear [134]. BCIs can improve QoL by maintaining or restoring communication, which is a prerequisite exercise of autonomy. On the other hand, there are concerns about potential social pressure on patients to use the technology and about ways in which BCIs could limit the patients’ control over communication. One question, for example, is how we can ascertain that observed (BCI-mediated) expressions coincide with users’ endorsed actions [135]. Does “Yes” always mean “Yes,” or is it sometimes a mistake by the BCI?

Finally, a related issue is the effect of BCIs on attribution of responsibility. In a study of stakeholders’ opinions on ethical issues related to brain–computer interfacing, most respondents agreed that BCI users are responsible for the executed actions and transmitted messages created with the aid of a BCI device [136]. This could expose users to responsibilities that are currently not well understood, both from a legal and ethical perspective [134].

#### Informed consent for BCI research and treatment

Ethical standards for research and clinical care require that a decision to undergo surgery to implant a BCI and then use the device should be voluntary and informed, and made by an individual with clear decision-making capacity [137]. However, each of the elements mentioned above—voluntariness, informed consent, capacity—is made more complicated in the context of later stage ALS. The decision to enroll in a trial of an implantable BCI, or to use a BCI as a therapeutic device in the future, must be well-informed. The risks, benefits, and alternatives need to be communicated to and understood by individuals contemplating an implantable BCI. This communication, and ensuring that it is well understood, is difficult given the technical complexity of the device and the accumulating yet still relatively limited safety data. Furthermore, unrealistic expectations may be engendered by inadequate attention to the limitations of current BCI technology among researchers and in the media. Ensuring that information on risks and benefits has been communicated effectively—asking individuals if they have understood presented information—can be especially challenging in the setting of severe communication impairment, especially if individuals are partially or completely locked-in [138–140] and will require carefully developed procedures [9, 141].

In addition to communication problems, decision-making in late-stage ALS raises issues of voluntariness and decisional capacity. The lives of people with ALS are typically marked by a growing web of dependencies on others, first with instrumental activities of daily living (e.g., transportation, financial management) and eventually with basic activities of daily living (e.g., feeding, bathing, toileting) [142, 143]. Each PALS usually becomes the center of a unique physical and social micro-environment. If an implantable BCI trial or therapy offers the prospect of changing the character or degree of dependency on others, a PALS may feel obligated to pursue a BCI [144]. Depending on the nature of this felt obligation, the voluntariness of the decision to have a BCI implanted may come into question. Conditions that co-occur with ALS, like frontotemporal dementia, hypoxia and hypercarbia from hypoventilation, dehydration, malnutrition, chronic pain, sleep deprivation, fatigue, depression, or (the side effects of) medications given to treat co-morbid conditions can also impair cognition [145]; thus, they may undermine the elements of decisional capacity (understanding, reasoning, appreciation, and choice [146]). One study of a large ALS clinic found that up to 20% of patients were only marginally capable or clearly incapable of treatment-related informed consent as measured by the MacCAT-T [147]. Taken together, these challenges to obtain valid informed consent for BCI research and treatment need careful case-by-case assessment of cognitive function and decision-making capacity by expert clinicians. Such assessments are also relevant for the research itself, as they will inform the research team about the potential capacity of the participant to learn to use a BCI. In addition, a clear understanding of a user’s cognitive function, placed in the context of their mastery of a BCI, will enhance understanding of the cognitive capacity needed to operate a BCI.

#### Access and continued use

Access to implantable neural devices in both research and clinical contexts raises important issues. In the research context, access to trials of BCI communication devices is limited. The technology is expensive and running the respective studies requires significant amounts of grant funding to cover the hardware, medical, and surgical costs related to the implantation, and personnel required for BCI training. In addition, the technical and clinical expertise to run trials is limited to academic medical centers, typically in urban areas; this often geographically limits the PALS who can participate. In addition, inclusion criteria for neural device trials or medical recommendation for an approved neural device can require stable and supportive social and familial environments; this criterion may exclude marginalized populations whose environments are shaped by lower resources. Racial disparities in the dissemination of DBS for Parkinson’s disease have been found in the US [148] and are a cautionary tale for future dissemination of BCIs. Also differences in educational opportunities and achievement may affect an individual’s familiarity with, and interest in, technology, and perhaps even the ability to master the use of a BCI.

Issues of access to neural devices can also extend past the conclusion of a BCI device trial [149–151]. International regulations such as the Declaration of Helsinki (Fortaleza Brazil, 2013) and the CIOMS guidelines (WHO, 2016) require that research sponsors and researchers make arrangements for participants who benefit from a research treatment, to facilitate continued access to the treatment, or provision of an equal alternative after a trial is completed. From a more clinical perspective, for PALS to eventually have access to implanted BCIs for communication, an important requirement is health insurance coverage of the device itself, of the medical procedures associated with implantation and of the AAC- or BCI-experts required for training and continued support [122]. This factor may represent yet another source of inequity, given differences between countries in the systems and rules for health insurance coverage of implanted neurotechnology. Although neurotechnology is still a relatively novel intervention in healthcare, lessons learned from more mature applications of neurotechnology (e.g., deep brain stimulation [152]) may help guide BCI clinical dissemination.

As noted, in-depth understanding of the supportive and prohibitive factors for adequate BCI performance is essential. Obtaining this knowledge will require extensive validation research in the daily-life settings of people with severe motor impairments. A thorough and iterative information and informed consent procedure must ensure that PALS participating in these studies do not have unrealistic expectations about BCI capabilities or about the chance that they experience benefit from their research participation [141]. Furthermore, these participants, and future users of clinically applied BCIs, will need to be prepared for the possibility that BCI performance may be affected by progressive disease, changes in the electrode–tissue interface, or plasticity affecting the neural signals used for BCI control.

Even when a device is scientifically or clinically effective, ethical issues still need attention. For instance, using a device may put extra demands on family or caregivers in terms of training, maintenance, trouble shooting or monitoring. Thus, a broader or more relational understanding of how devices affect agency and autonomy is warranted [153]. While a BCI may reduce certain vulnerabilities (e.g., being unable to communicate in emergent circumstances), it may also create new vulnerabilities (e.g., reliance on a battery, on a particular company that may run out of business, or on a research team or institution) [154]. Finally, a BCI device may be relied upon in circumstances in which miscommunication or malfunction could have devastating or irremediable consequences (e.g., incorrectly communicating a desire to withdraw treatment or request aid in dying) [139, 149].

### Need for a multidisciplinary approach towards clinical application of implantable BCIs

Since BCIs conceptually are a muscle-independent access method for augmentative and alternative communication technology [155], clinical and daily-life implementation may be driven by the same considerations that have been proposed within the wider field of assistive technology [156]. Their value for an individual will depend on his/her wishes and needs, disease stage, remaining capabilities, living environment, support system, and other factors. Furthermore, the value of an implanted BCI should be compared to the value of any muscle-based communication methods that the individual can still use. Because an implanted BCI entails surgery, additional ethical and medical factors should be considered as well, as detailed above.

Given the importance and complexity of the decision to adopt an implanted BCI, the decision should engage a multidisciplinary team that includes, besides a rehabilitation specialist or speech/language therapist, a neurosurgeon, a neurologist, an anesthesiologist, a psychologist, a social worker, an ethicist, the primary physician, and/or a neural engineer (cf. [157]). Standardizing and optimizing this multidisciplinary process may eventually require a dedicated BCI subspecialty in the field of rehabilitation, alternative and augmentative communication technology, and/or speech/language therapy [122]. A BCI clinical subspecialty may also contribute to an optimal user and caregiver training process and to optimal continued support during daily-life use. Furthermore, beyond the multidisciplinary team described here, the wider community of health care professionals who care for PALS should be well-informed about the potential risks, benefits, considerations, and procedures associated with implanted BCIs for PALS, and able to participate in ensuring effective clinical dissemination and use of these devices. Therefore, we strongly encourage comprehensive, vigorous, and ongoing discussions among BCI researchers, primary, secondary, and tertiary end users, other stakeholders, and all relevant clinical disciplines about *if*, *how, for whom,* and *when* implanted BCIs should be introduced in the multidisciplinary management of ALS. Hopefully, such discussion will develop basic principles before implantable BCIs become available for widespread dissemination and use, and will then continue to grow in sophistication and value as that use proceeds.

## Concluding remarks

Implantable BCIs show strong potential for improving and/or maintaining the quality of life of PALS. Nevertheless, a host of scientific, technical, medical, and ethical issues needs to be addressed before these devices can be offered as a clinical solution to the communication problems that PALS often encounter in late stages of the disease. Given the medical procedures involved in obtaining an implanted BCI, and the interaction these procedures may have with other aspects of the clinical management of ALS, we believe that researchers, clinicians, and other relevant stakeholders must combine their varied and complementary expertise regarding these issues and work together to ensure that the issues are addressed appropriately. Events organized by the International BCI Society (www.bcisociety.org) and groups such as NeuroAbilities (https://neuroabilities.org/) that supported the initial discussions of these authors and cross stakeholder lines, can provide a platform for discussions that bring together the many groups of BCI stakeholders and can promote the responsible development and implementation of this technology. In addition, significant dedicated effort and funding may enable the development of formalized recommendations for the clinical application of implantable BCIs.
